# Whole-Body Dose of Patients Treated With ZAP-X Intracranial Gyroscopic Stereotactic Radiosurgery

**DOI:** 10.7759/cureus.96093

**Published:** 2025-11-04

**Authors:** Georg A Weidlich

**Affiliations:** 1 Medical Physics, ZAP Surgical Systems, San Carlos, USA; 2 Medical Physics, National Medical Physics & Dosimetry Company, Inc., Palo Alto, USA

**Keywords:** in vivo, photon stereotactic radiosurgery, unintended dose, whole-body dose, zap-x® radiosurgery

## Abstract

During the delivery of radiosurgery treatments, a small dose is given unintentionally to the areas of the patient’s body outside the primary treatment volume. It is a common practice for radiotherapy platform developers to minimize the dose outside the primary radiation field by providing X-ray target shielding in the beam-defining collimator head of such systems. The amount of such doses was measured for the ZAP-X^®^delivery platform by designing a phantom-based set-up mimicking the patient and measuring the dose with an ionization chamber and OSLDs. Furthermore, during several clinical ZAP-X radiosurgery treatment deliveries, the dose received by patients was measured with film-based dosimeters. The results were determined as absolute values and as a percentage of the primary dose.

## Introduction

The delivery of radiation therapy in medicine has been accepted as one important pillar in the treatment of malignant and benign diseases. In addition to the benefits of radiation therapy, one unavoidable consequence is the delivery of a small amount of unintentional radiation to the patient’s body. This secondary radiation consists of X-ray target leakage and scatter radiation emanating from the treatment isocenter. Both types of radiation ultimately contribute to the dose received by the patient’s body [1].

Electrons are accelerated within the linear accelerator and focused onto the X-ray target, where they are converted by the Bremsstrahlung effect to photons. This conversion produces photons that are emitted in many directions, with their energy varying greatly depending on the interactions of the electron with the X-ray target material. While the most common direction of emission is forward along the beam path, some of the photons are scattered at large angles in directions that are considered not useful for treatment. The primary beam is collimated by heavy and dense tungsten photon jaws or stereotactic tungsten cones to deliver the prescribed dose to the target volume, while other beam components that are not used for treatment are typically shielded by the linear accelerator collimator housing. The amount of allowable leakage radiation through the collimator is stipulated by the International Electrotechnical Commission (IEC), which specifies the minimum amount of shielding required by a linear accelerator system that will limit the unattended patient dose to an acceptable level (IEC 60601-2-1) [2].

A new specialized device for radiotherapy was introduced in 2016 with the advent of the gyroscopic radiosurgery system called the ZAP-X® [3]. The ZAP-X was designed for dedicated use in intra-cranial stereotactic radiosurgery and employs a relatively low photon energy of 3 MV for optimized beam penetrative quality in the cranium. In addition, the collimator housing was designed to minimize those components of the radiation that reach the patient’s body at large distances from the target. The collimator consists of a massive tungsten shield and a collimator wheel of 15 cm in diameter, which is equivalent to five tenth-value layers (TVLs) [4] at 3 MV photon energy. Collimator sizes of 4-25 mm can be selected. This shielding will result in an X-ray target leakage of approximately 0.001% of primary radiation [5].

The other form of secondary radiation is due to the scatter radiation caused by the interaction of the primary beam with the tissues of the target volume. The mean intensity of such scatter radiation is approximately 0.1%, but varies greatly in intensity and energy.

For the ZAP-X, the energy of the X-ray target leakage is similar to the primary accelerating potential of 3 MV, while the scatter radiation is typically of much lower energy in the range of 100 keV to 200 keV. The combination of high-energy/low-intensity and low-energy/high-intensity radiation allowed the system to become self-shielded with relatively little shielding material embedded in the shells of the gyroscopic structure [6].

It is this unintended secondary radiation reaching the patient’s body unintentionally that is being investigated here.

## Technical report

**Figure 1:**
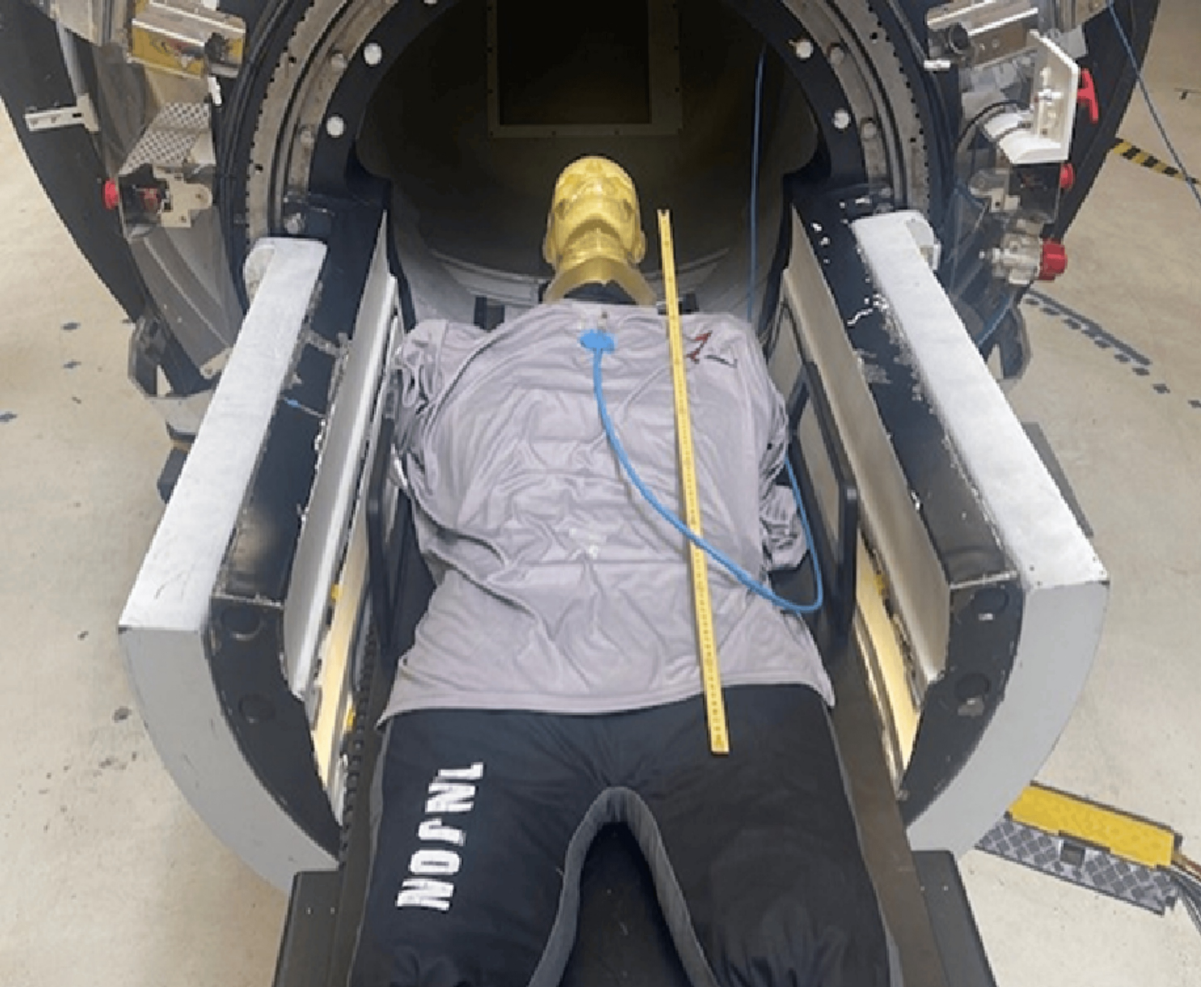
Phantom and test setup on the ZAP-X system

A phantom mimicking the patient’s body was constructed consisting of an anthropomorphic head phantom and tissue-mimicking body shape made of a water-equivalent microsphere material. The phantom is shown in Figure 1.

Two intracranial treatment plans were generated for the treatment of trigeminal neuralgia (TGN) and for a solitary metastasis, as indicated below. Each treatment plan consisted of a single isocenter, focusing on a target near the center of the cranium:

First, 8,000 cGy were planned to be delivered to a TGN target at the 80% prescription line. The number of beams was 151, and the plan used 21,244.3 monitor units (MU) and was delivered with a 4 mm circular collimator. The dose measurement was performed at 30 cm from the isocenter (thyroid position).

Second, 2,400 cGy were planned to be delivered to a solitary metastasis at the 80% prescription line. The number of beams was 151, and the plan used 6,368.7 MUs and was delivered with a 4 mm circular collimator. The dose measurement was performed at 30 cm from the isocenter (thyroid position).

Both treatment plans consisted of the largest possible spatial distribution of beams to mimic a typical clinical treatment plan. The smallest 4 mm collimator was used for both plans to generate a plan with a maximum number of MUs representing the worst-case scenario for this test.

**Figure 2:**
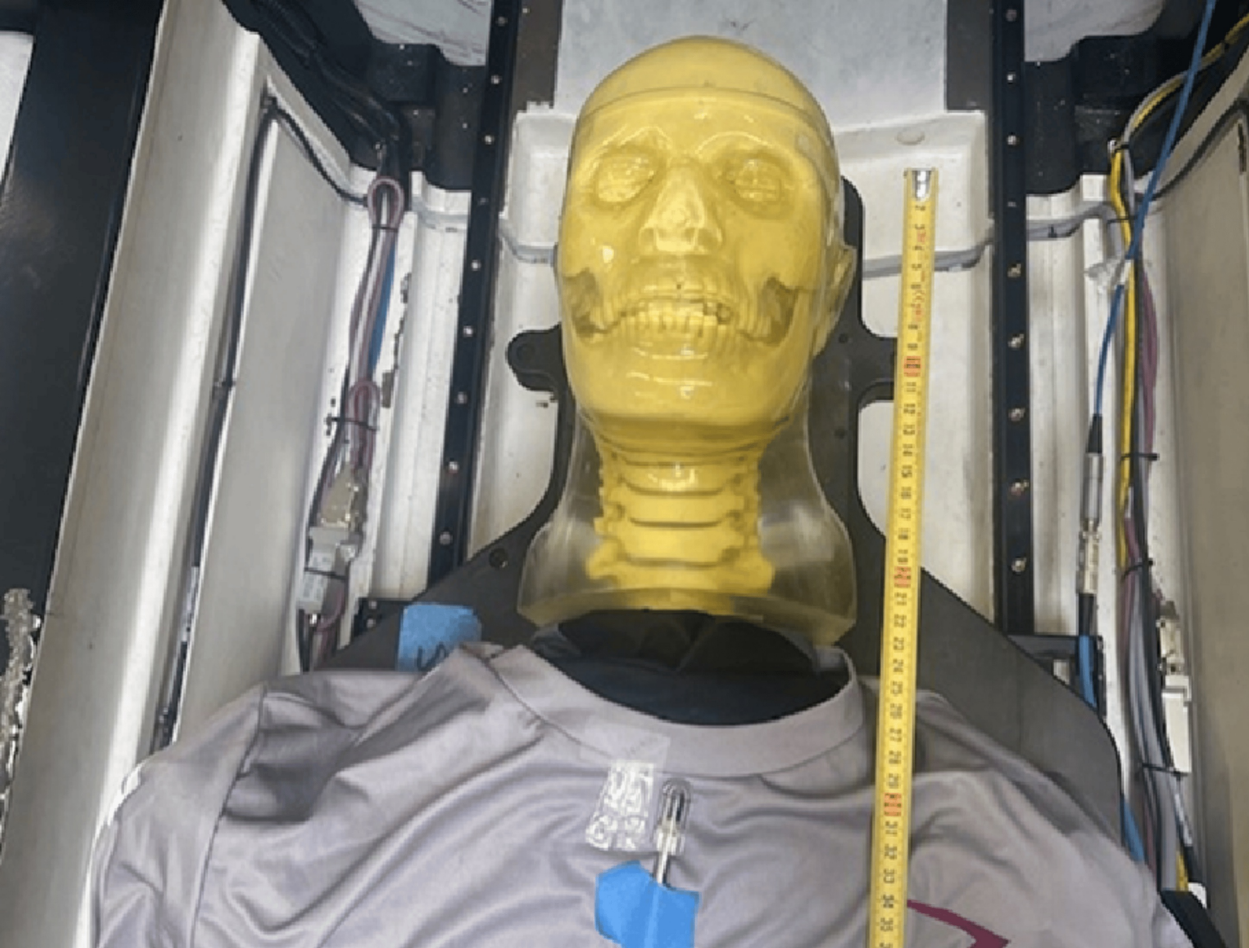
Measurement setup with the detector position

A PTW Semiflex 3D ionization chamber model number 31021 with a 0.07 cm^3^ active detection volume and a maximum build-up cap was placed at 30 cm distance from the isocenter at the approximate location of the thyroid on the surface of the phantom. The measurement setup is shown in Figure 2.

Using the onboard kilovoltage (kV) Gyroscopic Image Guidance system, the anthropomorphic head phantom was aligned to register the phantom with respect to the isocenter and to deliver the respective treatment plan. During this simulated treatment delivery, the dose received by the ionization chamber was measured after the chamber calibration for the ZAP-X 3MV primary beam was performed. The resulting data were recorded for each treatment delivery.

Results

The mean results of the phantom-based and in vivo patient-based measurements are shown in Table 1:

**Table 1:** Treatment plan parameters and measurement results of whole-body dose measurements The measured dose is shown as a mean value including one standard deviation. The uncertainty range of the measurements is determined by the reproducibility of the measurement setup and limiting parameters of the dosimetry system, as well as variations in patient body habitus and imaging frequency during intra-fraction IGRT (image-guided radiotherapy).

Treated target volume	Treatment plan parameters	Phantom
Trigeminal neuralgia	Prescription dose [cGy]	8,000
	Prescription isodose line	80%
	Number of beams	151
	Monitor units	21,244.30
	Collimator size [mm]	4
	# Isocenters	1
	Mean measured dose (mGy)	4.995 +/- 0.235
	Percentage of prescription dose	0.01%
Solitary brain metastasis	Prescription dose (cGy)	2,400
	Prescription isodose line	80%
	Number of beams	151
	Monitor units	6,368.70
	Collimator size (mm)	4
	# Isocenters	1
	Mean measured dose (mGy)	1.503 +/- 0.068
	Percentage of prescription dose	0.01%

## Discussion

The level of investigated doses is very small and on the order of doses allowable to be received by members of the public. Furthermore, the doses are much smaller than those received by other linear accelerator (Linac)-based radiotherapy delivery platforms.

This characteristic presents a significant advantage for pediatric treatments, with its patient population expected to have the longest post-treatment lifespan and associated risk of increased cancer incidence.

In addition, such low radiation leakage values theoretically enable the treatment of pregnant women. When projected to a typical isocenter to a uterus distance of 65 cm, even the relatively high whole-body radiation leakage dose of 5 mGy at 30 cm for the TGN case is expected to be reduced to 0.92 mGy, which is approximately 20% of the allowable fetal dose for the nine-month gestation period.

Albeit the differences in treatment planning and prescription dose, the global maximum dose for the phantom-based measurement setup was determined to be less than 5 mGy at a 30 cm distance from the isocenter, while the maximum dose received by patients during treatment was determined to be less than 3.34 mGy at 30 cm distance from the isocenter.

## Conclusions

For ZAP-X treatments, the doses received by areas of the patient body outside the treatment volume and at larger distances from the treatment isocenter were determined to be limited to 5 mGy. Among all Linac and external beam radiotherapy systems, the ZAP-X is expected to deliver the lowest such dose to the patient’s body.

Therefore, the ZAP-X system can be considered as one of the safest systems regarding the probability of secondary cancer incidence rate and cancer mortality rate.
