# Correction: Whole-Body Dose of Patients Treated With ZAP-X Intracranial Gyroscopic Stereotactic Radiosurgery

**DOI:** 10.7759/cureus.c419

**Published:** 2026-03-26

**Authors:** Georg A Weidlich

**Affiliations:** 1 Medical Physics, ZAP Surgical Systems, San Carlos, USA; 2 Medical Physics, National Medical Physics & Dosimetry Company, Inc., Palo Alto, USA

This article has been corrected at the request of the author to remove potentially misleading text as well as data from Table 1. A single treatment fraction is shown instead of the entire treatment course. It is the entire treatment course that represents the dose to the thyroid that is relevant to this article. Therefore, Table 1 was misleading as only a partial dose of the entire treatment course is represented.

